# Development of an Emulsion-Gel System Based on Corn Alcohol-Soluble Protein and Curdlan: Effects of Oil-to-Water Ratio and Shear Parameters

**DOI:** 10.3390/gels12050416

**Published:** 2026-05-10

**Authors:** Shijia Li, Chao Wu, Xiaojing Kang, Ran Wang, Qiang Cui, Yuyu Zhang, Mingkun Liu, Beibei Dou, Yang Liu, Han Chen

**Affiliations:** 1 Food Laboratory of Zhongyuan, China Agricultural University, Luohe 462000, China; lishijia@cau.edu.cn (S.L.); wuchao980103@163.com (C.W.); wangran@cau.edu.cn (R.W.); liumingkun0203@163.com.com (M.L.); doubeibei@zyfoodlab.cn (B.D.); liuyang@zyfoodlab.cn (Y.L.); 2 Department of Nutrition and Health, China Agricultural University, Beijing 100193, China; 3 Key Laboratory of Functional Dairy, Co-Constructed by Ministry of Education and Bejing Government, China Agricultural University, Beijing 100193, China; 4 FLZ-Sunjock Dairy Co. Joint Laboratory, Luohe 462000, China; tn2020036@zyfoodlab.com; 5 Mengniu Dairy (Jiaozuo) Co., Ltd., Jiaozuo 454002, China; 6 School of Food and Health, Beijing Technology and Business University, Beijing 100048, China

**Keywords:** zein, curdlan, emulsion gel, microstructure, stability

## Abstract

This study reports the fabrication of Pickering emulsion gels stabilized by zein and curdlan (CU) and systematically elucidates the regulatory mechanisms of oil fraction and shearing parameters (temperature and duration) on their microstructure, mechanical properties, and stability. Results indicated that excessive oil content triggered pronounced flocculation and structural collapse, primarily attributed to insufficient interfacial coverage and compromised network continuity. An optimal dense network with superior cohesiveness was established at a shearing temperature of 60 °C, effectively entrapping oil droplets within the continuous phase. Furthermore, extending the shearing duration enhanced long-term stability by reducing droplet size, yielding a maximum oil binding capacity (OBC) of 78.9%. These emulsion gels exhibited remarkable stability against diverse environmental stressors, including thermal treatments, pH variations, and freeze-thaw cycles. This work expands the application of protein-polysaccharide complexes in food colloid science and provides a theoretical foundation for the development of novel low-fat food formulations based on emulsion gel systems.

## 1. Introduction

Emulsion gels are semi-solid materials characterized by a three-dimensional network architecture derived from conventional emulsions, effectively integrating the functional attributes of both colloidal dispersions and gelled matrices [1]. These systems are generally categorized into emulsion-filled gels and emulsion-aggregated gels. In emulsion-filled gels, the continuous phase constitutes a coherent gel matrix wherein oil droplets are dispersed as discrete filler particles. Dickinson argues that these droplets can be further classified as either active or inactive fillers based on their interfacial interactions with the surrounding network [2]. Active fillers are mechanically anchored to the three-dimensional gel framework through covalent or non-covalent interactions [3], thereby reinforcing the structural integrity of the matrix [4]. Compared to traditional liquid emulsions, emulsion gels exhibit superior structural stability, enhanced oil-holding capacity, and distinct rheological responses under thermal or mechanical stress. Consequently, they hold significant potential for diverse applications, including fat replacement, active ingredient delivery, and the formulation of functional food products [5,6,7].

The fabrication strategies for Pickering emulsion gels primarily encompass physical, enzymatic, and chemical cross-linking approaches. In chemical methodologies, cross-linking agents such as formaldehyde and glutaraldehyde are frequently employed for industrial polymer synthesis; however, their inherent toxicity renders them unsuitable for food-grade applications [8]. Currently, salt-induced gelation and enzymatic cross-linking are the predominant techniques utilized within the food sector, yet they are often constrained by prolonged gelation durations and high operational costs [9]. Consequently, simple physical fabrication methods have gained increasing prominence. Research has demonstrated that thermal treatment can effectively trigger gelation by exposing cryptic binding sites within protein molecules, thereby facilitating the formation of robust networks via hydrophobic interactions, hydrogen bonding, and disulfide linkages [10,11]. Notably, plant-derived proteins, as natural biopolymers, exhibit superior emulsifying properties, interfacial activity, and biodegradability [12]. Leveraging the synergistic interactions between plant proteins and polysaccharides to construct composite emulsion gels has emerged as a pivotal research direction in food structural design.

However, the inherent structural rigidity of most plant proteins, stemming from the sequestration of hydrophobic residues within their interior, limits their interfacial efficacy in stabilizing Pickering emulsion gels [13]. Consequently, the transition from simple “ingredient filling” to sophisticated “functional mimicry” can be realized through the development of protein-polysaccharide complex systems. Previous studies have demonstrated that polysaccharides can mitigate the coalescence and aggregation of protein-stabilized droplets by screening electrostatic charges or enhancing the viscosity of the continuous phase, thereby reducing molecular collision rates [14]. Curdlan (CU), a unique thermo-gelling polysaccharide, forms a thermo-reversible “low-set” gel sustained by weak hydrogen bonding upon heating to approximately 55 °C [15]. This biopolymer can establish a dense and homogeneous hybrid network with proteins through a combination of hydrogen bonding, hydrophobic associations, and physical entanglements. While existing literature predominantly focuses on protein modification and polysaccharide concentration, the impact of processing parameters on the structural evolution and performance of these systems remains insufficiently explored, with most studies concentrating on shear rate. A systematic investigation is needed to elucidate the mechanisms by which shear duration influences the preparation and internal structure of Pickering emulsion gels.

Therefore, in this study, a Pickering emulsion-gel system was constructed solely through shear forces by preparing a complex of Zein and curdlan. The resulting systems were comprehensively characterized in terms of droplet size distribution, oil binding capacity (OBC), rheological behavior, textural attributes, and multi-environmental stability. Furthermore, the microstructural evolution and molecular mobility were elucidated using Confocal Laser Scanning Microscopy (CLSM) and Low-Field Nuclear Magnetic Resonance (LF-NMR), providing a systematic understanding of the regulatory mechanisms underlying oil fraction and shearing parameters (temperature and duration). This work offers critical processing insights and a theoretical framework for the design of novel, protein-polysaccharide-based low-fat food formulations.

## 2. Results and Discussion

### 2.1. Effect of Oil Volume Fraction on the Properties of Emulsion Gels

#### 2.1.1. Microstructure

**Figure 1:**
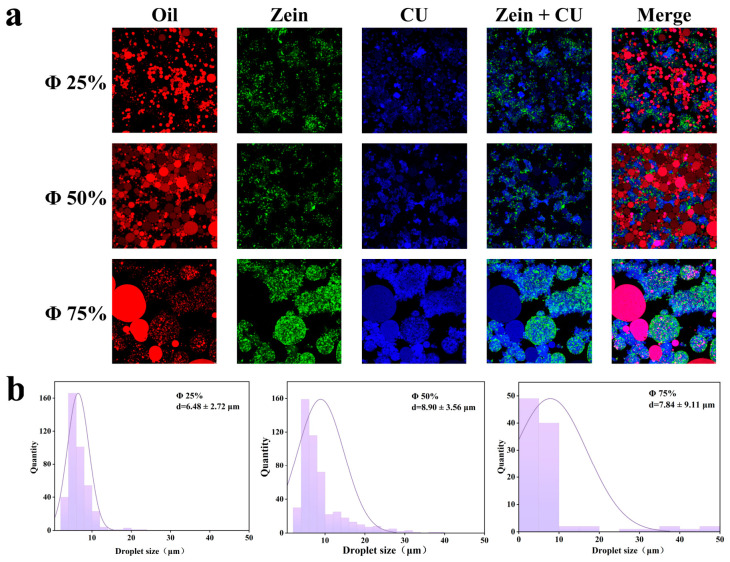
Confocal laser scanning microscopy (CLSM) images and droplet size distributions of emulsion gels at different oil-to-water ratios. (**a**): CLSM (The red signal indicates oil, the green signal indicates protein, and the blue signal indicates Curdlan.); (**b**): droplet size distribution. The oil-to-water ratios, from left to right, are 25%, 50%, and 75%.

Confocal laser scanning microscopy (CLSM) allows for direct visualization of a sample’s microstructure and distribution. Figure 1a shows CLSM images at different oil-to-water ratios, where red, green, and blue correspond to the spatial distribution characteristics of the oil phase, Zein, and Cu, respectively. The three-dimensional spatial barrier formed by Zein nanoparticles in conjunction with CU effectively suppresses the flocculation of oil droplets in the emulsion system [16]. The oil droplets, acting as the dispersed phase, are embedded within the protein-polysaccharide network structure. At lower oil content, there is sufficient space in the continuous phase to anchor the oil droplets within the gel network. As the oil content increases, the number of oil droplets filling the gel network rises, and the distance between droplets further decreases, thereby enhancing the driving force for coalescence. At a 25% oil phase ratio, the oil phase is uniformly dispersed in the continuous phase as small, uniform oil droplets with no obvious aggregation or coalescence. Zein is evenly distributed in the aqueous phase, and some green fluorescence is observed on the surface of the red-fluorescent oil droplets, indicating that protein particles adsorb at the oil-water interface to form an interfacial film. The composite (CU + Zein, blue-green overlay channel) exhibits a uniform interpenetrating network structure. When the oil phase ratio was increased to 50%, the number of oil droplets increased significantly, and the size of some droplets grew; however, the system maintained good overall dispersion. The composite network structure was filled with oil droplets, reducing porosity. The adsorption of Zein on the oil droplet surfaces increased, making the interfacial film denser and effectively suppressing the aggregation and coalescence of the oil droplets. At an oil phase ratio of 75%, the oil droplet size increased significantly, and noticeable aggregation and coalescence occurred. The system transitioned from “oil droplet dispersion” to a “continuous oil phase”, resulting in a significant decline in structural stability.; The study by Wu et al. [17] showed that when the oil content increased to 60%, the higher oil content led to flocculation of the droplets within the emulsion-gel system, causing the droplet size to increase markedly, accompanied by phenomena such as destabilization. The study by Lim et al. [18] similarly found that when the oil content exceeded 50%, phase separation occurred within the system, making it impossible to successfully prepare an emulsion-gel; furthermore, emulsion-gels prepared using single protein particles lacked sufficient structural stability. This indicates that the Zein-Cu composite system can effectively anchor droplets within the continuous phase and suppress their movement.

#### 2.1.2. Droplet Size Distribution

The droplet size distribution of the emulsion-gel was determined by analyzing CLSM images using ImageJ (Fiji) software. Droplet size is one of the most important parameters in evaluating emulsion-gel systems; it is closely related to characteristics such as stability. Furthermore, changes in the oil-to-water ratio alter the number of particles adsorbed at the oil-water interface, thereby affecting droplet size [19]. The droplet size and distribution at different oil-to-water ratios are shown in Figure 1b. As the oil content increases, the droplet size exhibits a trend of first increasing and then decreasing, rising from 6.48 ± 2.72 μm at 25% oil content to 8.90 ± 3.56 μm at 50% oil content. This is primarily due to the increased oil content leading to a greater number of oil droplets in the system and reduced distances between them, making collisions between droplets more likely, leading to droplet coalescence and the formation of larger droplets. It is worth noting that when the oil content increased to 75%, although the average diameter decreased slightly, the standard deviation was extremely large. Furthermore, CLSM images revealed that the droplet sizes within the system were highly uneven, indicating that the increased number of oil droplets resulted in more frequent encounters between droplets, and the intensified coalescence led to the continuous formation of large droplets. On the other hand, the increased oil content requires Zein-CU particles to cover a larger oil-water interface, potentially leaving insufficient particles to cover the entire interface. The droplet size distribution curve provides a better assessment of the uniformity of droplets within the system. Generally, the ideal curve exhibits a narrow, single-peak distribution. As shown in Figure 1b, the distribution curve becomes broader as the oil content increases, and this may increase the structural heterogeneity of the system, thereby thermodynamically increasing the likelihood of droplet coalescence.

#### 2.1.3. Oil Binding Capacity

**Figure 2:**
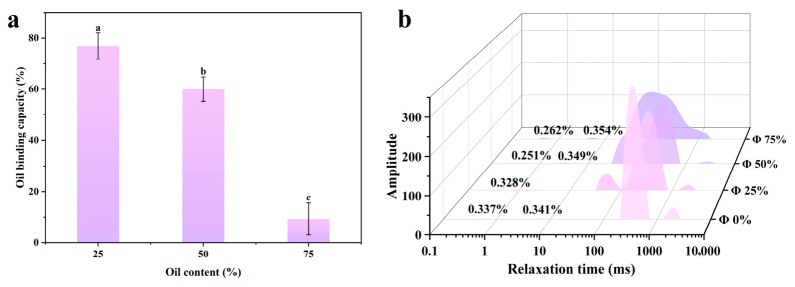
Oil binding strength of emulsion gels at different oil-to-water ratios (**a**) and T_2_ spectra in low-field NMR (LF-NMR) (**b**). (a–c) represent significant differences between different emulsions at the *p* < 0.05 level.

Oil binding capacity is one of the key indicators for evaluating the density of the internal network of emulsion gels. Generally, a loose, porous three-dimensional network structure has a wide and uneven pore size distribution, which leads to a decrease in the physical retention of oil [20]. The oil binding efficiency of emulsion gels at different oil content levels is shown in Figure 2a. As the oil content increases, the oil binding efficiency gradually decreases, dropping significantly from 76.91% at 25% oil content to 9.28% at 75% oil content. This is primarily due to the fact that, under conditions of high oil content, the coverage of particles at the oil-water interface is insufficient, resulting in significant differences in curvature between droplets. Thermodynamically, this increases the tendency for droplets to coalesce under the influence of Laplace pressure, thereby reducing the oil retention capacity. On the other hand, an increase in the oil phase proportion leads to dense droplets within the system that disrupt the original three-dimensional network structure of the continuous phase, thereby reducing its ability to confine the oil. This is consistent with the previous CLSM results, where an increase in oil content causes droplet coalescence.

#### 2.1.4. Low-Field Nuclear Magnetic Resonance (LF-NMR)

Low-field nuclear magnetic resonance (LF-NMR) is commonly used to analyze the distribution and migration patterns of water in hydrophilic colloid-protein complex systems (NMR). In low-field NMR T_2_ relaxation spectra, three distinct characteristic peaks appear, corresponding to three states of water: bound water (T_2b_), semi-bound water (T_21_), and free water (T_22_). As shown in Figure 2b and Table 1, the addition of oil shortens the relaxation times at T_21_ and T_22_ (shifting them to the left). Compared to the control group without oil (0%), the water distribution in the oil-containing system is more stable, and the degree of water confinement is higher. As the oil content increases, the A_21_ value of the gel system gradually rises, reflecting that the filling effect of the oil converts free water in the composite system into immobilized water with lower mobility. Furthermore, the proportion of A_21_ in the system is significantly higher than that of A_2b_, which is mainly attributed to the Zein-CU complex binding the aqueous or oil phase through non-covalent interactions, whereas A_2b_ is primarily due to the strong binding of water molecules by the gel. Therefore, the A_2b_ content remains relatively stable in emulsion-gel samples with different oil contents.

#### 2.1.5. Rheological and Textural Properties

**Figure 3:**
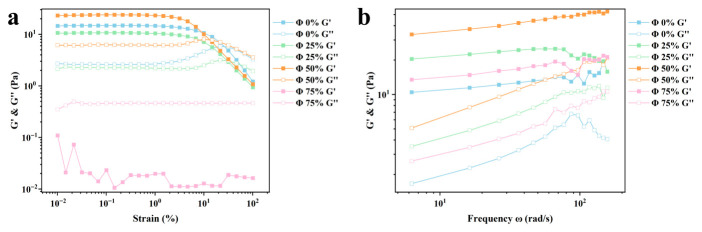
Strain-frequency (**a**) and frequency-frequency (**b**) scans of emulsion gels at different oil-to-water ratios.

The linear viscoelastic region (LVR) of the Pickering emulsion gels was determined via strain-amplitude sweeps. The LVR represents the critical strain range where the sample structure remains intact and the stress is proportional to the applied strain, thereby reflecting the mechanical robustness of the internal network. Generally, a wider LVR is indicative of a more stable and resilient gel framework [21]. As illustrated in Figure 3a, all samples exhibited a distinct LVR at strains ranging from 0.01% to 1.0%.

Notably, the storage modulus (G′) reached its maximum at an oil fraction (Φ) of 50%. This enhancement is attributed to the oil droplets acting as “active fillers” that are mechanically anchored to the Curdlan (CU) network, thereby reinforcing the overall structural integrity of the composite system. In contrast, at an oil content of 75%, both G′ and the loss modulus (G″ dropped sharply, with the system transitioning to a liquid-like behavior (G″ > G′). This structural impairment potentially stems from the excessive displacement of the continuous phase by the dispersed oil, coupled with insufficient interfacial coverage by the Zein-CU complexes, which likely induced significant droplet coalescence. These rheological observations are highly consistent with the CLSM results, which revealed a fragmented network at high oil fractions, as well as the previously noted reduction in oil binding capacity.

The results of the frequency scan are shown in Figure 3b. It can be seen that G′ is consistently greater than G″ for all emulsion gel samples, indicating an elastic-dominated behavior. This can be attributed to the continuous phase network formed by the protein-polysaccharide complex, which restricts the flow of lipids and thereby promotes the formation of the gel network structure. At the same time, both G′ and G″ gradually increase with increasing frequency, exhibiting slight frequency dependence, indicating that the samples are more resistant to deformation at higher frequencies. This may be because any structural changes within the emulsion gel do not occur within the time span of a single oscillation [22,23]. No yield point (G′ < G″) was observed even under high-frequency scanning, indicating that the gel network maintains its structural integrity under high shear rates without undergoing a gel-sol transition [24]. The G′ of the samples with added oil phase was significantly higher than that of the control group without added oil phase, and G′ gradually increased with increasing oil content, which is consistent with the results of Wu et al. [17]. This is mainly due to the droplets within the system becoming closer together, enhancing the interactions between droplets, while the adsorption of lipids onto protein nanoparticles strengthens the interactions within the composite structure, thereby improving the mechanical strength of the gel [25]. In contrast, G′ decreased significantly at 75% oil content, likely due to structural disruption within the sample under high-shear conditions. Combined with the large oil droplets observed via CLSM, this indicates that the internal structure of the system at higher oil contents is highly fragile and insufficient to withstand high-frequency deformation.

The textural properties of the emulsion gel at different oil addition ratios are shown in Table 2. As can be seen from the table, as the oil content increases, the hardness of the system decreases significantly from 24.33 ± 2.08 g (without oil) to 11.00 ± 1.00 g (25% oil content), reaching a minimum of 2.67 ± 0.58 g at 75% oil content. This is primarily because the structural strength of the system is mainly provided by the continuous phase; an increase in the oil content further weakens the cross-linking effect of the curdlan within the system. At the same time, liquid oil droplets, acting as the dispersed phase, weaken the strength of the gel network at higher concentrations. Cohesiveness, on the other hand, exhibited a trend of first increasing and then decreasing. At lower oil contents, the protein-polysaccharide complex successfully encapsulated the oil droplets within the network; a moderate increase in oil droplets further acted as “active fillers” to enhance the internal bonding strength of the system, demonstrating better synergy. However, when the oil content reached 75%, the excessive oil disrupted the continuous-phase network formed by the curdlan, causing the internal structure of the system to become more loosely organized. Chewiness showed no significant change between 0% and 50% oil content but decreased significantly at 75% oil content, producing a lubricating effect, consistent with the previous results.

#### 2.1.6. Stability

**Figure 4:**
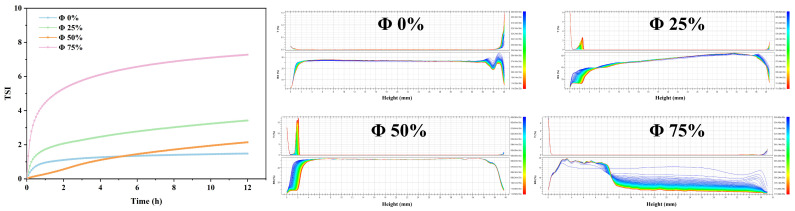
Effect of different oil-to-water ratios on the gel emulsion’s instability index (TSI).

A laser scanning device was used to evaluate the resistance to phase separation of emulsion gels under gravitational forces at different oil-to-water ratios, and the kinetic stability index (TSI) values were calculated [23]. The results are shown in Figure 4. A steeper slope of the TSI curve and a higher TSI value indicate that the system is less stable. As shown in the figure, the maximum slope and TSI value (7.5 ± 0.54) were observed at an oil content of 75%, while the lowest TSI value (2.1 ± 0.64) was recorded at an oil content of 50%, indicating the highest stability. This is closely related to the network structure in the continuous phase and the interactions between oil droplets. The backscattered light image reflects the emulsion separation and flocculation of the emulsion-gel system over a 12-h period [26]. As oil is added, oil droplets rise under the influence of gravity. The least amount of rise occurs at an oil content of 50%, primarily because the oil droplets are firmly anchored in the continuous phase at this point, as verified by the oil binding strength discussed earlier. When the oil content is 25%, the backscattered light intensity shows an upward trend as the sample position changes, indicating the heterogeneity of this emulsion structure. At fat concentrations of 25% and 75%, distinct emulsion stratification was observed. This effect can be attributed to two factors: (1) at low fat concentrations, oil droplets cannot fully fill the continuous phase; under the dehydration-condensation effect of the protein-polysaccharide composite system and the influence of gravity, the continuous phase settles to the bottom; (2) at high oil content, the particle concentration in the continuous phase is insufficient to completely cover the significantly enlarged oil-water interface, resulting in free oil. This result is consistent with the images obtained from laser confocal microscopy.

### 2.2. Effect of Different Shear Conditions on Gel Emulsions

#### 2.2.1. CLSM

**Figure 5:**
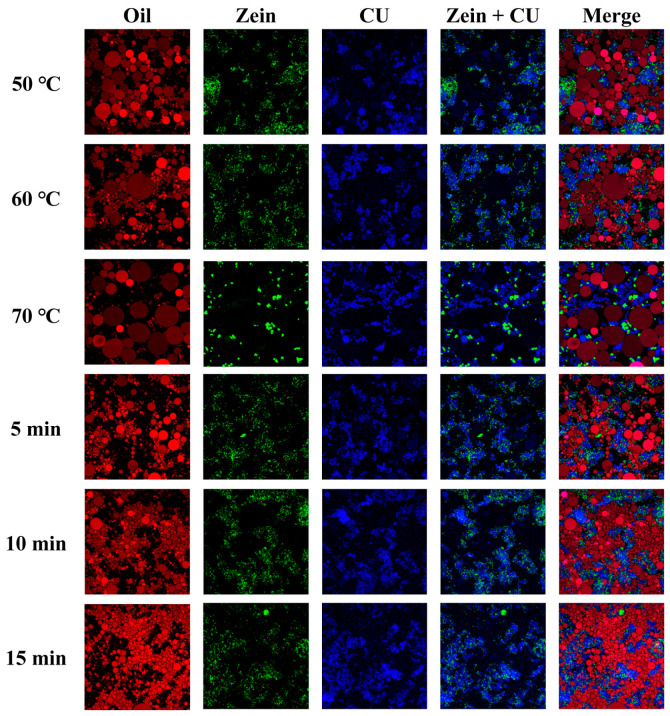
CLSM images of emulsion gels under different shear conditions. The red signal indicates oil, the green signal indicates protein, and the blue signal indicates Curdlan.

The processing method is one of the key kinetic parameters that govern the properties of emulsion gels. The CLSM images of emulsion gels prepared under different shear conditions are shown in Figure 5. At a shear temperature of 50 °C, the resulting droplet sizes are uniform with no significant coalescence. The interfacial film formed by Zein-CU further inhibits the formation of large oil droplets, while CU constitutes a stable network framework for the continuous phase, and the distribution of Zein within the continuous phase further enhances its structural integrity. As the temperature rises to 60 °C, the uniformity of droplet size within the system decreases, and significant coalescence occurs. Additionally, Zein is distributed more uniformly within the CU-structured continuous phase; this may be due to the looser arrangement of CU molecules at higher temperatures, which reduces the viscosity of the continuous phase. When the temperature was further increased to 70 °C, the droplet size in the system increased significantly, and aggregation between droplets became more pronounced. This may be due to the significant condensation of the CU network structure at high temperatures, the disruption and reorganization of intermolecular forces within the gel network [15], and the thermal denaturation and aggregation of proteins, leading to the destabilization of the interfacial film. Furthermore, under high-temperature conditions, Brownian motion among molecules in the system intensifies, causing more frequent collisions between droplets and facilitating the formation of larger droplets, which leads to system destabilization.

As the shear duration increases, the droplet size becomes smaller and more uniform. This is primarily due to the deformation and fragmentation of droplets under continuous shear, which simultaneously promotes particle adsorption at the oil-water interface and enables tighter packing. At the same time, after 5 min of shear, the interfacial film is unable to tightly encapsulate the oil droplets; the droplet size distribution is uneven, with noticeable large droplets present, and the continuous phase contains numerous network pores, indicating that the shear energy input at this stage is insufficient. As the time is further extended, the large oil droplets break down further, and the droplet size becomes more uniform. When the shear duration increases to 15 min, the droplet size reaches its minimum and becomes highly uniform, exhibiting a state of high-density packing. At this point, the shear energy has achieved an extremely high level of dispersion in the oil phase, and the composite continuous-phase network formed by Zein and CU tightly encapsulates the oil droplets, preventing coalescence between them.

#### 2.2.2. Droplet Size Distribution

**Figure 6:**
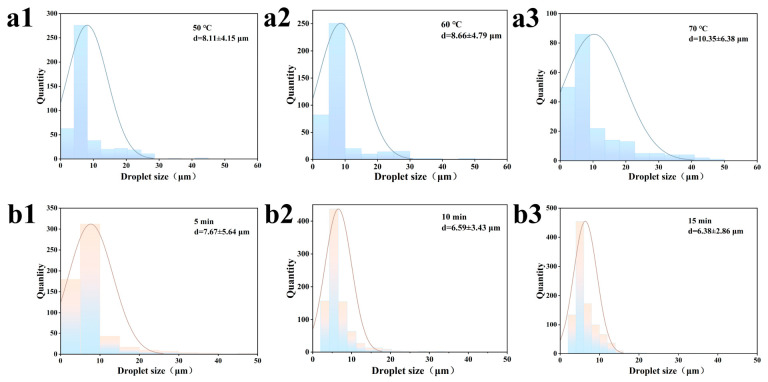
Droplet size and distribution of emulsion gels under different shear conditions. (**a1**–**a3**) correspond to temperatures of 50, 60, and 70 °C, respectively, while (**b1**–**b3**) correspond to shear durations of 5, 10, and 15 min, respectively.

The droplet sizes and distributions at different shear temperatures are shown in Figure 6(a1–a3) and Table 3. As the temperature increases, the droplet size gradually increases and the distribution curve broadens, rising from 8.11 μm at 50 °C to 11.44 μm at 70 °C. This phenomenon is primarily due to the increased fluidity of the oil phase under heating conditions, intensifying Brownian motion between droplets and making collisions and coalescence more likely, resulting in larger droplet sizes, as confirmed by CLSM images. Concurrently, the molecular structure of proteins is disrupted under heating conditions, exposing hydrophobic groups. Under the influence of hydrophobic interactions, proteins come into closer proximity and aggregate [1]. Some protein particles detach from the interfacial film, exposing oil droplets and leading to further aggregation.

As shown in Figure 6(b1–b3) and Table 3, as the shear duration increases, the droplet size becomes smaller and more uniform, decreasing from 7.67 μm at 5 min to 6.38 μm at 15 min, indicating that droplet coalescence has been suppressed. Generally speaking, as shear duration increases, the probability and frequency of large droplets being broken up also increase. This helps reduce droplet size and improve dispersion, facilitating the formation of uniformly sized droplets and thereby enhancing stability. However, the stabilization of droplets by particles is not an instantaneous process. During the initial stage of shearing, the surface area of the newly formed oil-water interface increases significantly, and the particles cannot immediately cover the entire interface. Under continuous shearing, the particles have ample time to diffuse, adsorb, and rearrange. Only after the particles have completely covered the interface can the droplets avoid coalescence due to the physical barrier effect.

#### 2.2.3. Oil Binding Capacity

**Figure 7:**
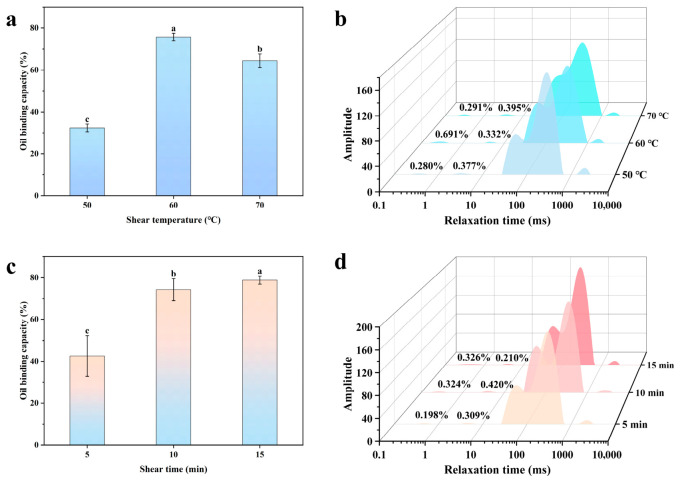
Oil binding capacity of emulsion gels under different shear conditions ((**a**): shear temperature; (**c**): shear duration) and T_2_ spectra in low-field nuclear magnetic resonance (LF-NMR) ((**b**): shear temperature; (**d**): shear duration). (a–c) represent significant differences between different emulsions at the *p* < 0.05 level.

Figure 7a shows the oil binding capacity of emulsion gels prepared at different shear temperatures. As the oil content increases, the oil binding capacity first rises and then falls. At a shear temperature of 50 °C, the oil binding capacity was lowest at 32.38 ± 1.85%; when the temperature was increased to 60 °C, it reached a maximum of 75.76 ± 1.72%. When the temperature was further increased, the oil-binding capacity decreases rather than increases, indicating that the composite gel network exhibits the strongest binding capacity for oil at 60 °C. This may be because Zein is a thermally induced gel-forming polysaccharide; at 50 °C, its molecular solubility and degree of hydration are low, making it difficult to form an effective continuous-phase network. When the temperature rises to 60 °C, Zein begins to swell and increases the viscosity of the system, enabling it to better trap oil droplets. At the same time, Zein particles in the continuous phase can completely cover the surface of the oil droplets, forming a stable interfacial film that restricts the mobility of the liquid oil [27].

The oil-binding capacity of the emulsion gels prepared under different shear durations is shown in Figure 7c. It can be seen that the oil-binding capacity gradually increases with prolonged shear duration, rising significantly from 42.56 ± 9.75% at 5 min to 78.86 ± 1.99% at 15 min. This phenomenon can be attributed to the fact that prolonged shearing sufficiently reduces the size of oil droplets and improves their distribution within the composite gel, allowing the interfacial film to fully spread between the aqueous and oil phases and enhancing the gel network’s ability to bind oils. On the other hand, prolonged shearing provides the particles with sufficient kinetic energy for adsorption, forming a dense and robust interfacial film that effectively resists droplet coalescence induced by Laplace pressure and generates strong steric hindrance between droplets, thereby enhancing the oil binding capacity.

#### 2.2.4. Low-Field Nuclear Magnetic Resonance (LF-NMR)

The LF-NMR results at different shear temperatures are shown in Figure 7b and Table 4. T_2_ < 10 ms corresponds to bound water, which is tightly bound to the CU-Zein gel network via strong hydrogen bonds and exhibits extremely low mobility. As the shear temperature changes, A_2b_ shows no significant variation, indicating that the proportion of bound water remains essentially stable, suggesting that the core water-binding capacity of the polysaccharide-protein network does not undergo drastic changes with temperature. The range 10 ms < T_2_ < 100 ms corresponds to semi-bound water filling the network pores. When the shear temperature was increased from 50 °C to 60 °C, A_21_ increased significantly; when the temperature was further raised to 70 °C, the A_21_ and A_22_ peaks were not completely separated, indicating that the semi-bound water had almost disappeared. The T_2_ > 100 ms range corresponds to free water. Compared to 70 °C, the water distribution within the system is more stable at 50 °C and 60 °C. This is primarily because at 60 °C, the intact protein-polysaccharide interpenetrating network provides ample pore space for water and lipids, accommodating a large amount of semi-bound water while effectively trapping free water. When the temperature exceeds 70 °C, Zein undergoes irreversible thermal denaturation, leading to protein aggregation, the rupture of the interfacial membrane, and the destruction of the hydrogen bond network in the CU by excessive heating, which alters the gel network structure. This causes the semi-bound water (A_21_) originally confined within the pores to be released, transforming into highly mobile free water.

As shown in Figure 7d and Table 3, the values of A_2b_ and T_2b_ for Sample A_2b_ did not change significantly with increasing shear duration, indicating that the water bound by strong hydrogen bonds within the CU gel network is not affected by shear duration. Within the range of 10 ms < T_2_ < 100 ms, A_21_ first increased and then decreased with increasing shear duration, reaching a maximum value (31.47%) at 10 min. When T_2_ > 100 ms, the free water in the system first decreases and then increases with increasing shear duration, with A_22_ reaching its minimum at 10 min. This is likely because the network structure in the continuous phase is uniformly distributed at this point, adsorbing a large amount of free water into the micropores. At 15 min, A_22_ shows a rebound, yet T_22_ reaches its lowest value. This was primarily because the droplets had shrunk to their smallest size at this point, resulting in a significant increase in interfacial area and a tighter packing of Zein particles at the oil-water interface. This highly dense interfacial adsorption likely produced a certain degree of steric hindrance, causing some water molecules that were originally weakly bound to the protein layer to be released into the continuous phase which is consistent with the high OBC results.

#### 2.2.5. Rheological and Textural Properties

**Figure 8:**
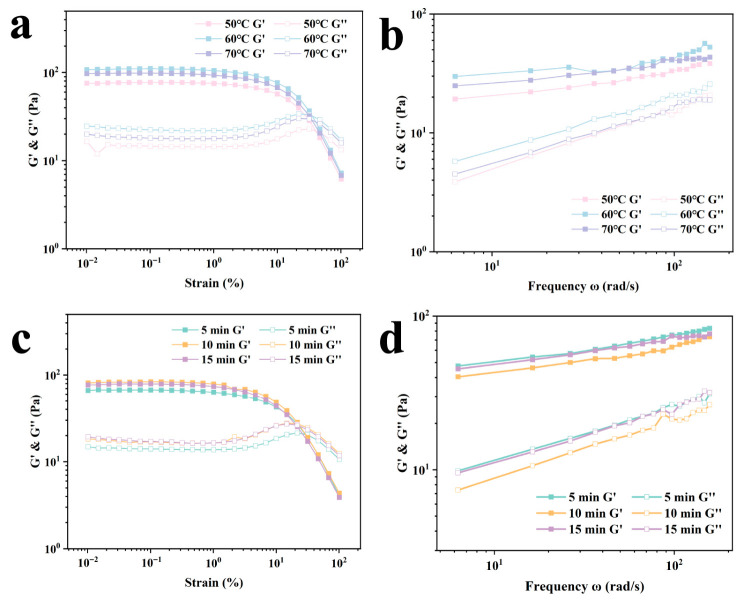
Strain scans ((**a**): shear temperature, (**c**): shear duration) and frequency scans ((**b**): shear temperature, (**d**): shear duration) of emulsion gels under different shear conditions.

Figure 8a,b show the rheological results of the emulsion gel at different shear temperatures. As can be seen from the figures, G′ is consistently greater than G″, indicating that elasticity dominates the emulsion gel system. The lowest G′ value is observed at 50 °C, which is primarily due to reduced cross-linking between CU units at lower temperatures; the network structure has not fully developed, resulting in a weaker gel framework. At a shear temperature of 60 °C, G′ is highest across the entire frequency range, indicating a more compact gel network structure within the system. This is likely because the curdlan has undergone better hydration and swelling at this temperature, and the Zein particles have formed the most stable interfacial film at the oil-water interface, which synergizes with the continuous phase network. However, when the temperature increased to 70 °C, G′ decreased. This may be related to the thermoreversible properties of the curdlan. CLSM images show that, at this point, the green signal no longer continuously envelops the edges of the red oil droplets but is instead scattered throughout the continuous phase. This may be due to the destabilization of the interfacial film or the rearrangement of protein particles.

As shown in Figure 8c, G′ is at its lowest under a shear duration of 5 min. This indicates that, within a short period, the oil droplets are not sufficiently broken down and the Zein particles fail to achieve complete coverage at the oil-water interface; under high shear stress, this is insufficient to support the structural stability. As shown in Figure 8d, G′ reaches its highest value at a 5-min shear duration. This may be due to the aggregation of incompletely disrupted droplets; the stronger attractive and interactive forces between droplets increase the structural strength within the system. Research by Wu et al. [28] similarly demonstrated that G′ increases due to lipid aggregation.

As shown in Table 5, the highest hardness was observed at 50 °C, reaching 7.67 ± 0.58 g. This may be attributed to the lower degree of swelling and hydration of the curdlan in the continuous phase at lower temperatures; these partially expanded, relatively hard polysaccharide particles act as inactive fillers within the system and exhibit a certain degree of resistance to macroscopic compression. Cohesiveness, however, reached its maximum at 60 °C. This is likely because at this temperature, CU begins to gradually transition from a particulate state to a network structure, and the oil droplets stabilized by Zein can form stronger molecular entanglements and interactions with the continuous-phase network formed by CU. As shear duration increases, hardness first decreases and then increases. This is because the shear energy applied at 5 min did not completely disrupt the original gel network of CU, which retained a relatively high hardness. The rebound in hardness, cohesiveness, and chewiness observed at 15 min is primarily due to the prolonged shear duration, which caused the droplets within the system to become smaller and more tightly packed, resulting in the strongest network structure.

#### 2.2.6. Stability

**Figure 9:**
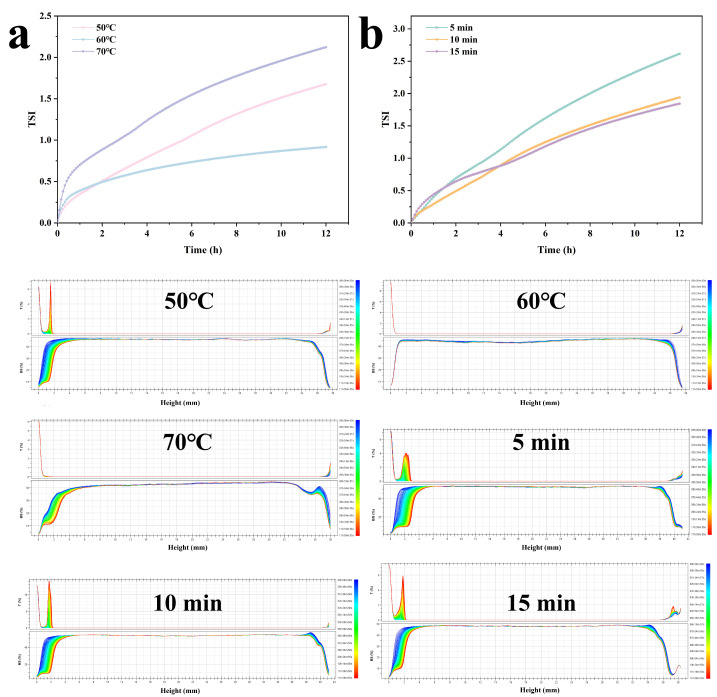
Effect of different shear rates (**a**) and shear durations (**b**) on the gel-emulsion instability index (TSI).

As shown in Figure 9a, the effect of different shear temperatures on the system’s stability, from strongest to weakest, is: 60 °C > 50 °C > 70 °C. This finding is consistent with previous results: at lower temperatures, protein molecules lack sufficient mobility, and their ability to adsorb lipids in an ordered manner is insufficient, leading to an uneven distribution of the interfacial film; however, excessively high temperatures cause irreversible protein aggregation, destroying the interfacial film structure. At 60 °C, the interfacial flexibility of Zein reaches equilibrium with the hydration and swelling of CU. Protein particles form the tightest interfacial film at the interface, effectively inhibiting droplet coalescence through a physical barrier. At the same time, the viscoelasticity and cohesiveness of the continuous phase are at their peak, effectively trapping oil droplets and slowing their rise due to gravity.

The TSI curves of the emulsion gel under different shear durations are shown in Figure 9b. As the shear duration increases, the stability peaks at 15 min. This is primarily attributed to the refinement of droplets and the dense packing of particles at the interface; this Pickering mechanism provides a mechanical barrier that inhibits the movement of oil droplets. The corresponding backscatter light spectra show that the backscatter light intensity at the bottom of the emulsion gradually decreases with different shear durations, indicating clarification at the bottom of the emulsion. Compared to the curves at t = 0, the degree of clarification at the bottom gradually decreases with increasing shear duration, and the height of the clarified region decreases from 6 mm to 3 mm. At the top of the emulsion, the backscattered light intensity (BS%) decreased slightly over time. At 180 min, a small amount of fat floated to the top of the emulsion, while the middle section indicated that no flocculation or coalescence of droplets had occurred within the system.

### 2.3. Environmental Stability

To further investigate the potential applications of Pickering emulsion-gel systems in real-world food industry processes such as processing, storage, and transportation, the preparation process for Pickering emulsion-gels was further optimized, and the fat content was reduced to meet low-fat requirements. An emulsion-gel product was developed using a fat content of 15%, a shear temperature of 60 °C, a 25-min shear duration, to simulate a low-fat processed cheese product. The system’s stability under heat treatment, pH changes, and freeze-thaw cycles was evaluated based on two parameters: fat binding capacity and instability index, with untreated samples serving as the control group.

**Figure 10:**
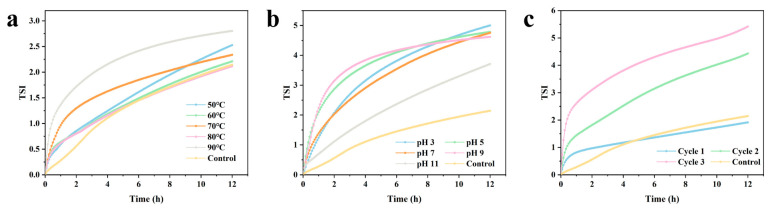
TSI curves of the emulsion gel under different temperatures (**a**), pH levels (**b**), and freeze-thaw cycle numbers (**c**).

As shown in Figure 10a, as the temperature increased from 50 °C to 90 °C, the TSI index exhibited a trend of first decreasing and then increasing. At 60 °C and 80 °C, the TSI values were 2.2 and 2.1, respectively, representing extremely low levels. When the temperature exceeded the gelation threshold of CU, CU transformed from a low-temperature gel into a high-temperature irreversible gel. This sudden increase in physical cross-linking strength further anchors Zein at the interface, hindering the coalescence of oil droplets [15]. Although the TSI rises to 2.8 at 90 °C due to partial protein denaturation, the overall structure remains intact. Wu et al. [17] found that the structure of the emulsion gel became more stable after moderate heating. Heating caused partial denaturation of the protein particles adsorbed at the oil-water interface. At the same time, due to subunit dissociation and partial unfolding of the globular proteins, buried hydrophobic sites were exposed, and the hydrophobic groups of the proteins were brought to the protein surface. Subsequent hydrophobic interactions caused the proteins to come closer together and aggregate, thereby improving their performance at the oil-water interface and promoting protein cross-linking.

The pH environment of processed cheese typically ranges between 5.5 and 6.5; therefore, the stability of the emulsion under acidic conditions determines its scope of application. Within the pH range of 3–11, the TSI value decreased from 5.0 to 3.7; at pH 5 and 7—which are close to the neutral and slightly acidic environments of cheese—the TSI was 4.8 and 4.7, respectively. The emulsion’s stability decreased slightly under strongly acidic conditions due to the influence of protein isoelectric points; however, within the mildly acidic range commonly used in cheese processing, the sample exhibited a high level of stability. The TSI of the fresh sample was 2.1; after one freeze-thaw cycle, the TSI rose only to 1.9, indicating that the initial freeze-thaw had a negligible effect on the system’s microstructure. When the number of cycles increased to three, the TSI rose to 5.4. This reflects the damage caused by repeated ice crystal growth to the anti-squeeze structure. The high-density composite network formed by the emulsion gel effectively mitigates the coalescence of oil droplets induced by ice crystals during the first freeze-thaw cycle, and this demonstrates that the emulsion-gel system exhibits excellent resistance to agglomeration and oil separation during cold-chain storage, which is crucial for extending the shelf life of oil-containing foods. Its high stability under various environmental conditions indicates that the emulsion-gel system can serve as a fat substitute in products such as ice cream and acidic dairy beverages, while also withstanding pasteurization processes.

## 3. Conclusions

In this study, a Pickering emulsion-gel system stabilized by a complex of Zein and curdlan was developed, and the mechanisms by which component ratios and processing conditions regulate its microstructure and stability were systematically elucidated. The results indicate that oil content is a key factor determining the stability of the emulsion-gel. When the oil content is too high, the gel network in the continuous phase loses its ability to constrain oil droplets, leading to increased droplet coalescence. Processing conditions, by regulating the size of internal droplets and the viscosity of the continuous phase, can influence the stability of the emulsion-gel system. Research has confirmed that shear duration is a key parameter in regulating interfacial kinetic equilibrium; by imparting sufficient kinetic energy to interfacial particles to overcome the adsorption energy barrier, it induces the interfacial film to evolve from a loosely distributed state to a highly dense, packed state. The combination of confocal laser scanning microscopy (CLSM) and low-field nuclear magnetic resonance (NMR) further confirmed that the dense network structure in the continuous phase is crucial for achieving high oil-binding capacity and long-term kinetic stability, and is capable of withstanding adverse factors encountered in actual food processing. The system’s stability within a pH range of 5–7, as well as its resistance to heating and freeze-thaw cycles, demonstrate its technical feasibility in real food matrices such as low-fat cheese. This study harnesses shear energy to overcome the resistance to particle rearrangement at interfaces, thereby achieving a transition from “composition-based filling” to “function-based simulation.” This clean-label preparation process provides safer, lower-cost guidance for industrial applications and offers important process guidance and theoretical foundations for the development of low-fat foods based on protein-polysaccharide systems.

Although this study has elucidated the physical regulation mechanisms of emulsion-gel systems influenced by preparation processes, future research should evaluate the kinetics of lipid release through in vitro simulated digestion experiments and further elucidate the dynamic stability patterns of composite interfacial films under complex processing conditions, thereby providing more in-depth theoretical guidance for their application in targeted delivery systems for functional foods.

## 4. Materials and Methods

### 4.1. Materials

Zein was purchased from Sigma-Aldrich (St. Louis, MO, USA). Curdlan (CU) was obtained from Shandong HaiAoSi Bio-Technology Co., Ltd. (Zibo, China). Absolute ethanol (analytical grade) was supplied by Shanghai Macklin Biochemical Co., Ltd. (Shanghai, China). Sodium hydroxide (NaOH), hydrochloric acid (HCl), and trisodium phosphate were purchased from Sinopharm Chemical Reagent Co., Ltd. (Shanghai, China). Fluorescent dyes, including Nile Red and Nile Blue A, were acquired from Sigma-Aldrich (St. Louis, MO, USA). Deionized water was used for all solution preparations and experiments. Corn oil was purchased from Yihai Kerry Group (Beijing, China).

### 4.2. Preparation of Zein-Curdlan (CU) Complexes

Following the method described by Zhong et al. with minor modifications, corn alcohol-soluble protein nanoparticles were prepared using the anti-solvent precipitation method [29]. Briefly, 2.0 g of Zein was dissolved in 100 mL of a 70% (*v*/*v*) aqueous ethanol solution and stirred overnight to ensure complete dissolution. This solution was then added dropwise into 100 mL of deionized water under magnetic stirring at 800 rpm for 1 h. Subsequently, ethanol was partially removed using a rotary evaporator (RE-5000, Baer Instrument and Equipment Co., Ltd., Xi’an, China) at 46 °C for 20 min until approximately half of the initial volume had evaporated. The final concentration of the ZNP dispersion was adjusted to 2 wt%.

Curdlan (CU) dispersion was prepared by dispersing 2.0 g of CU powder in 200 mL of deionized water (pre-adjusted to pH 8.0). The mixture was stirred at 60 °C for 2 h to achieve uniform dispersion.

To form the Zein-CU complexes, the ZNP dispersion was slowly introduced into the CU solution at 40 °C to maintain a Zein:CU mass ratio of 1:1. The pH of the resulting mixture was precisely adjusted to 8.0 using 0.1 M HCl or trisodium phosphate solutions. The composite system was then stirred at 800 rpm for 1 h to yield the Zein-CU complex dispersion. The total concentration of the complex particles was adjusted to 1.5 wt%.

### 4.3. Preparation of Pickering Emulsion Gels

The composite solution was mixed with corn oil at different oil-to-water ratios, with oil contents of 0.25, 0.50, and 0.75 (at 60 °C and a shear duration of 5 min), using a shear mixer (F6/10 F01320021, Jingxin Industrial Development Co., Ltd., Shanghai, China) at temperatures of 50 °C, 60 °C, 70 °C, and 80 °C (oil content 0.5, shear duration 5 min), and at 1000 rpm for 5 min, 10 min, and 15 min (oil content 0.5, temperature 60 °C) to obtain emulsion-gel systems.

### 4.4. Confocal Laser Scanning Microscopy (CLSM)

The microstructure of the emulsion gels was visualized using a confocal laser scanning microscope (TCS SP8, Leica, Wetzlar, Germany) according to the method described by Cui et al. with minor modifications [16]. To distinguish the different components within the matrix, a triple-staining procedure was employed. Specifically, 1 mL of the emulsion gel was aliquoted and thoroughly mixed with 40 µL of Nile Red (1 mg/mL in ethanol), 40 µL of Nile Blue (1 mg/mL in ethanol), and 40 µL of Calcofluor White to fluorescently label the oil phase, proteins, and polysaccharides, respectively. The stained samples were placed on concave microscope slides and covered with coverslips. Observation was performed using excitation wavelengths of 543 nm (Nile Red), 633 nm (Nile Blue), and 405 nm (Calcofluor White).

### 4.5. Droplet Size Analysis

The droplet size distribution and mean diameter of the emulsion gels were quantified through digital image analysis of the CLSM micrographs using ImageJ software (Version 1.53, National Institutes of Health, Bethesda, MD, USA). Briefly, the original micrographs were converted into 8-bit grayscale images. A binary thresholding procedure was then applied to effectively segment the dispersed oil phase from the continuous gel matrix. To resolve overlapping or aggregated droplets, a watershed algorithm was employed for automated segmentation. Using ImageJ software, the surface area (*A*) of at least 500 individual droplets from multiple representative microscopic fields was measured. The equivalent spherical diameter (*d*) of each droplet was calculated according to Equation (1):

(1) $$d=2\times \sqrt{A/\pi }$$

### 4.6. Oil Binding Capacity (OBC)

The oil binding capacity (OBC) of the emulsion gels was determined according to the method of Wu et al. [30] with slight modifications. Briefly, approximately 1 g of the emulsion gel was accurately weighed into a 5 mL centrifuge tube and subjected to centrifugation at 7155 g for 15 min at 25 °C. Following centrifugation, the tubes were inverted for 30 min to allow the released free oil to drain. Any remaining mobile oil was carefully removed by blotting with filter paper. The OBC was calculated gravimetrically using Equation (2):

(2) $$OBC\left(\%\right)=\left(1-\frac{M_{1}-M_{2}}{M}\right)\times 100\%$$

where, *M*_1_ is the mass measured before centrifugation (g), *M*_2_ is the mass measured after centrifugation (g), and *M* is the mass of fat in each emulsion gel sample (g).

### 4.7. Low-Field Nuclear Magnetic Resonance (LF-NMR)

At room temperature, 3–5 g of emulsion-gel samples were placed in a nuclear magnetic resonance (NMR) spectrometer (NMI20-040V-I03100129, Suzhou, China). The instrument was set to the following parameters: operating frequency of 20 MHz, low-frequency magnetic field strength of 0.5 ± 0.03 T, with 5000 echoes and 4 repetitions. For the data obtained from the measurement of emulsion gel samples in each group, the T2 values were analyzed using the Karl-Purser-Mebom-Gill sequence. Simultaneously, the iterative reconstruction technique (SIRT) algorithm was used to construct the inversion decay curves. The spectra obtained from the emulsion gel samples in each group were normalized based on the weight of the respective samples.

### 4.8. Rheological Measurements

Following the experimental method described by Wu et al. [17] with minor modifications, the rheological properties of the emulsion-gel samples were measured using a rotational rheometer (MCR 302, Anton Paar, Graz, Austria). A 25-mm-diameter plate was used, with a gap set to 1 mm. Before each measurement, the sample was allowed to equilibrate at 25 ± 0.1 °C for 2 min to ensure thermal stability and eliminate residual mechanical stress. To determine the linear viscoelastic region (LVR), amplitude sweep tests were initially performed at a fixed frequency of 1 Hz, with the strain increased from 0.01% to 100%. Subsequently, frequency sweep tests were conducted over an angular frequency range of 1 to 100 rad/s at a constant strain of 0.1% (selected within the LVR). The storage modulus (G′) and loss modulus (G″) were recorded as functions of strain or frequency. Data acquisition and analysis were performed using the integrated rheometer software.

### 4.9. Texture Profile Analysis (TPA)

The textural attributes of the emulsion gels were evaluated using a texture analyzer (TA. XT Plus, Stable Micro Systems, Godalming, UK) equipped with a 7.5-mm diameter cylindrical probe (P/7.5), following the method described by Li et al. [31] with minor modifications. Prior to analysis, each sample was meticulously prepared into a uniform rectangular cuboid (1.5 × 1.5 × 0.8 cm). A double-cycle compression test was performed with the following instrumental settings: pre-test, test, and post-test speeds were all maintained at 1.0 mm/s, with a trigger force of 2.0 g. Each specimen was compressed to a depth of 4 mm. A dwell time of 15 s was maintained between the two compression cycles to allow for partial structural recovery. Textural parameters, including hardness, cohesiveness, and chewiness, were recorded and processed using the Exponent 4.0 software. All measurements were conducted in triplicate to ensure reproducibility.

### 4.10. Stability Analysis (Turbiscan Stability Index)

The physical stability of the emulsion gels was quantitatively evaluated using a multiple light scattering analyzer (Turbiscan Tower, Formulaction, Toulouse, France) [32]. Freshly prepared samples were transferred into specialized cylindrical glass cells and scanned at 25 °C for 12 h at 5-min intervals. The instrument monitors the variations in backscattering (ΔBS) or transmission (ΔT) profiles along the sample height as a function of time, which reflects microstructural changes such as droplet migration or aggregation. The overall kinetic stability was characterized by the Turbiscan Stability Index (TSI), calculated using the integrated software according to Equation (3):

(3) $$TSI=\sqrt{\frac{\sum_{i=1}^{n}{\left(x_{i}-x_{BS}\right)}^{2}}{n-1}}$$

where *n* represents the number of scans, *x_i_* denotes the average backscatter intensity per minute during the measurement process, and *x_BS_* is the average value of the sum of *x_i_*.

### 4.11. Environmental Stability

To assess the feasibility of the Zein-CU emulsion gels for practical food applications, their stability was evaluated under various thermal, pH, and freeze-thaw conditions.

Thermal stability: Freshly prepared emulsion gels were transferred into hermetically sealed glass vials and incubated in a temperature-controlled water bath at 50, 60, 70, 80, and 90 °C for 15 min. Subsequently, the samples were immediately quenched in an ice-water bath to reach room temperature. The stability index (TSI) of the gels before and after thermal treatment was measured and compared.

pH stability: The pH of the freshly fabricated emulsion gels was adjusted to values ranging from 3.0 to 11.0 using 0.1 M HCl or NaOH solutions.

Freeze-thaw stability: The samples were subjected to cryogenic storage at −20 °C for 20 h, followed by thawing at room temperature until both the oil and aqueous phases were completely liquefied. This process was repeated for up to three successive freeze-thaw cycles.

### 4.12. Statistical Analysis

All experiments were conducted at least in triplicate, and the data are expressed as the mean ± standard deviation. Statistical significance was evaluated by one-way analysis of variance (ANOVA) followed by Duncan’s multiple range test using SPSS software (Version 27.0, IBM Corp., Armonk, NY, USA). Differences were considered statistically significant at a confidence level of 95% (*p* < 0.05). All graphical illustrations and curve fitting were performed using Origin 2021 software (OriginLab Corp., Northampton, MA, USA).

## Figures and Tables

**Table 1:** Relaxation times and peak proportions of Pickering emulsion gel systems prepared with different oil-to-water ratios.

Simple	T_21_ (ms)	T_22_ (ms)	A_21_ (%)	A_22_ (%)
Φ 0%	-	333.12	-	94.65
Φ 25%	51.11	333.12	11.48	85.36
Φ 50%	51.11	333.12	29.13	69.19
Φ 75%	90.48	-	99.38	-

**Table 2:** Texture characteristics of Pickering emulsion gel formulations prepared with different oil-to-water ratios.

Simple	Hardness (g)	Cohesiveness	Chewability
Φ 0%	24.33 ± 2.08 ^a^	0.24 ± 0.03 ^c^	21.67 ± 4.73 ^a^
Φ 25%	11.00 ± 1.00 ^b^	0.35 ± 0.02 ^b^	19.67 ± 4.04 ^a^
Φ 50%	6.00 ± 1.00 ^c^	0.56 ± 0.02 ^a^	19.33 ± 2.08 ^a^
Φ 75%	2.67 ± 0.58 ^d^	0.15 ± 0.06 ^d^	5.33 ± 0.58 ^b^

Note: Data are expressed as the mean ± standard deviation. ^a–d^ indicate significant differences between samples (*p* < 0.05).

**Table 3:** Droplet sizes of emulsion-gel samples under different preparation processes.

Simple	Droplet Size (µm)
Φ 25%	6.48 ± 2.72 µm
Φ 50%	8.90 ± 3.56 µm
Φ 75%	7.84 ± 9.11 µm
50 °C	8.11 ± 4.15 µm
60 °C	8.66 ± 4.79 µm
70 °C	10.35 ± 6.38 µm
5 min	7.67 ± 5.64 µm
10 min	6.59 ± 3.43 µm
15 min	6.38 ± 2.86 µm

Note: Data are expressed as the mean ± standard deviation.

**Table 4:** Relaxation times and peak contributions of Pickering emulsion gel systems prepared under different shear conditions.

Simple	T_21_ (ms)	T_22_ (ms)	A_21_ (%)	A_22_ (%)
50 °C	54.78	270.49	24.01	73.69
60 °C	57.47	333.12	27.32	70.42
70 °C	-	310.78	-	98.29
5 min	58.73	289.94	26.27	72.10
10 min	58.73	357.08	31.47	66.99
15 min	54.79	252.35	24.18	74.22

**Table 5:** Texture Properties of Pickering Emulsion Gel Formulations Prepared with Different Oil-to-Water Ratios.

Simple	Hardness (g)	Cohesiveness	Chewability
50 °C	7.67 ± 0.58 ^a^	0.44 ± 0.02 ^c^	16.33 ± 2.30 ^a^
60 °C	6.00 ± 1.00 ^b^	0.56 ± 0.02 ^ab^	19.33 ± 2.08 ^a^
70 °C	6.33 ± 0.58 ^b^	0.51 ± 0.03 ^b^	16.00 ± 2.00 ^a^
5 min	6.00 ± 1.00 ^c^	0.56 ± 0.02 ^a^	19.33 ± 2.08 ^a^
10 min	5.67 ± 0.58 ^c^	0.48 ± 0.04 ^b^	12.67 ± 2.52 ^b^
15 min	7.33 ± 0.58 ^b^	0.50 ± 0.04 ^ab^	16.00 ± 1.00 ^ab^

Note: Data are expressed as the mean ± standard deviation. ^a–c^ indicate significant differences between samples (*p* < 0.05).

## Data Availability

The raw data supporting the conclusions of this article will be made available by the authors on request.

## Abbreviations

The following abbreviations are used in this manuscript:

CU	Curdlan
